# Physical Changes Affecting the Teeth of the Young

**Published:** 1881-10

**Authors:** A. J. Douds


### PHYSICAL CHANGES AFFECTING THE TEETH OF THE YOUNG.

READ BEFORE THE NORTHERN OHIO DENTAL ASSOCIATION BY A. J. DOUDS, D. D. S.

May 11th, 1881.

In the wide range of ailments to which the human body is subject none are of more interest than those presented in what is denominated caries of the teeth, and of all the different phases under which this disease is found no observing dentist will have failed to observe the marked tendency to rapid destruction so frequently found in the teeth of the young.

Even in childhood in many instances scarcely has the little pearl escaped from the gum before the destructive influence is found at work, and so general it may be that all local treatment directed to their preservation seems hopeless, and the only treatment promising the slightest prospect of relief must be of a general character.

It is the purpose of this brief paper to point out some of the causes of the very common tendency to disease and loss of a set of organs so vital to health and comfort during the periods of childhood, youth, and adolescence, but more especially at an age varying from ten to eighteen years.

Perhaps the most mortifying failures to which every dentist must plead more or less guilty, have been experienced right here, although honest effort accompanied by the most earnest solicitude may only have resulted in failure, and the conviction has been forced upon the mind that something more than the artistically executed operations upon which we so highly pride ourselves is required.

That there exists a greater liability to decay and loss of the teeth at certain periods of life will be readily recognized when we allude to absorption of the alveolar process, and consequent loss of the teeth in advanced age. The peculiar liability to decay during the periods of gestation and lactation, and to what it is especially desired to call attention, the destructive agencies at work during the periods referred to, viz.: youth and adolescence.

That a thorough comprehension of the nature and causes of disease of the teeth is just as essential to the dentist in the practice of his profession as it is for the practitioner of medicine in his more varied duties is so apparent, that to maintain this assumption no argument is needed.

It is not proposed here to discuss the influence of heredity, although Tomes, Wedl, and other eminent writers, attach to it importance of a primal character.

The pathological conditions commonly presented, and with which doubtless all are more or less familiar, are ropy, viscid, salivary, and mucuous secretions presenting a strong acid reaction. The teeth in many cases being covered with a dark, or greenish coating, adhering with great tenacity, the gums turgid and secreting a serous fluid, and upon raising the free edge of which, there will be found an entire absence of the coating referred to.

These symptoms are not, however, invariably present, as there may be an entire absence of the coating and but slight irritation of the margin of the gums, but invariably accompanied by a more or less hyper sensitive condition of dentine, as the character of the decay may determine. The symptoms of a general nature, accompanying and characteristic, can only be alluded to incidentally.

The dental organs should not be regarded as isolated portions of the economy, upon which the various evolutions in the process of growth development, nutrition, and changes of maturity can have no influence, but realizing the intimate relationship existing between all parts of the economy through the medium of the nerves and their connection with the great sympathetic system, we can more readily comprehend the mutual dependence existing between the teeth and the organs concerned in the nutrition of the body.

As the rapidity of the destruction of the teeth will depend not only upon the energy of the agent, but their texture; we may say in turn that their texture will depend largely upon their nutrition during the period of their development.

It is interesting at this point to observe the influence of the premature loss of the deciduous teeth, as well as that of the diseases of childhood, in interfering with the normal process of nutrition, and the consequent imperfect development of the permanent set, thus predisposing them to disease, and their liability to destruction becomes greater when during the periods referred to. Youth and adolescence characterized by evolution of the sexual organs, and by those general accompanying constitutional changes which constitute puberty, and during which periods, in order to the maintainance of the integrity and perfect development of every organ, there is imparatively demanded an *excess* of *constructive* over *destructive* change.

Should the great demand for functional activity not be maintained by a perfect nutrition, exhaustion of vital force will inevitably follow, and whatever greatly exhausts organic nervous power, predisposes to and occasions disease, so modifying the secretions; changing them from their normal standard, as in the oral secretions, as to serve as an exciting cause of caries.

As there can be at this period no state of inertia, the ever active never slumbering antagonistic forces are stimulated, and as Goethe so aptly expresses it, "Nature knows no pause in progress and development, and attaches her curse on all inaction."

In the breaking down of the vital powers, there is an illustration afforded even in active life of the great law of chemical affinity, acids are formed, which combining with the alkaline basis entering into the structure of the teeth, ammonia is set free, which being thrown off through the respiratory organs, instead of being eliminated by the kidneys, their natural channel, gives rise to the feted breath so frequently met with.

During this period is displayed, rather the exercise of the formative power, than the building up of the nervous and muscular vigor that the vital forces are expended, as Carpenter remarks, "it is familiar to every one that during the rapid growth of a young person, his nerve muscular energies are usually feeble and his powers of endurance brief."

That the liability to decay during this period is greater in females than males, will, it is believed, be sustained by the observation of all whose attention may have been directed to the matter, and why this is so becomes a subject of great interest.

Mr. Carpenter states that "there is no obvious difference in the nervous systems of the two sexes, save in the inferior size of the cerebral hemispheres," and there being no apparent structural difference upon which to ground a solution of the cause, the greater exhaustion of vital force consequent upon the more striking constitutional changes taking place in the female, together with her greater emotional nature, with its well kno wninfluence in modifying the secretions, seem not to fail more particularly in those of a highly nervous organization to effect such a morbid influence upon the oral secretions as to readily account for this greater tendency to decay.

Should the facts then, warrant in the foregoing conclusions, it will be evident that an intelligent comprehension of the pathological conditions, their immediate and remote causes would, it may be reasonably be inferred, enable us to so modify the course of disease, and if not in every case permanently arrest, at least greatly retard the progress of decay.

But in order to arrive at definite conclusions and results, the necessity for exact and comprehensive observation becomes imperative, for no knowledge is gained by intuition, and no opinion based upon more impirical practice can have the weight of well established facts.

After having studied the various phases of temperament, etc., such a course of treatment as will tend to restore the normal condition of an *excess* of *constructive* over *destructive* change, will afford gratifying results.

To this end, sound advice addressed to the mind rather then medicines to the stomach, coupled with a diet at once wholesome, nutritious, and *largely nitrogenous* with such tonics as special cases may require, may usually be relied upon to produce such changes for the better as will not only have earned for us the gratitude of an appreciative patient, but in the lesson of encouragement realize the gratifying consciousness that we have not labored in vain.

